# Trends in Internet Infrastructure Development and Online Health Use in China: 10-Year Descriptive Longitudinal Study

**DOI:** 10.2196/86714

**Published:** 2026-03-23

**Authors:** Chaoqun Wu, Yan Qiu

**Affiliations:** 1 Ningbo Medical Center Lihuili Hospital Ningbo, Zhejiang China; 2 First Affiliated Hospital Zhejiang University Hangzhou, Zhejiang China

**Keywords:** internet, internet trends, online health, online health users, China

## Abstract

**Background:**

Despite the rapid expansion of internet infrastructure and digital health initiatives in China, there remains a lack of longitudinal, nationally representative analyses that track the concurrent development of general internet access and the specific adoption of online health services over the past decade. Understanding these parallel trends is crucial for evaluating the reach and equity of the ongoing digital health transformation.

**Objective:**

The aim of this study was to describe decade-long trends in internet infrastructure development and online health service adoption in China through a comprehensive secondary analysis of nationally representative, publicly available survey data from 2014 to 2025.

**Methods:**

Data were retrieved from the official website of the China Internet Network Information Center for the period from June 2014 to June 2025. The study was conducted in 31 provinces, autonomous regions, and municipalities, excluding Hong Kong, Macau, and Taiwan. The participants were citizens aged 6 years and older who had a telephone or mobile phone. The China Internet Network Information Center conducted a stratified 2-stage sampling survey using a computer-aided telephone access system.

**Results:**

From 2014 to 2025, an increasing trend was observed in the number of internet users and the internet penetration rate in China. It also showed an upward trend in the number of internet users, both in urban and rural areas. A consistent increasing trend was detected in the number of mobile internet users. In contrast, desktops and laptops showed a declining trend. The number of online health users in China showed a V-shaped change from 2020 to 2025. In June 2025, the total number of online medical users reached approximately 393 million, representing 35% of all internet users.

**Conclusions:**

This decade-long observational study demonstrates sustained and significant growth in internet access across China, accompanied by a substantial rise in online health service adoption. A notable V-shaped trajectory in online health use emerged after 2020, indicative of a rapid COVID-19 pandemic–driven acceleration followed by market consolidation. The converging trends of near-universal smartphone-based access and the massive popularity of mobile-centric services, such as short videos, have fundamentally reshaped the digital landscape. Consequently, the findings suggest that for digital health strategies to achieve broad impact, policymakers and health care providers could consider prioritizing the integration of health promotion and services into existing high-penetration mobile platforms and communication formats that the population already uses daily.

## Introduction

With the rapid development of the economy and internet technology in China, the internet has become deeply embedded in the social fabric, serving as a vital platform for diverse activities. At the end of 2022, there were 3.53 billion mobile Internet of Things terminal connections in China, reflecting the extensive infrastructure supporting the Internet of Things [1]. Concurrently, the health care sector has undergone a significant digital transformation. Following the formal regulation of internet hospitals by the National Health Commission in 2018, these platforms have proliferated, providing essential services for consultation, chronic disease management, and health education [2,3]. This transformation aligns with global trends in digital health integration and has been accelerated by specific national policies aimed at promoting “internet and health care” [4]. Research has begun to document the positive impacts of such digital health interventions, including improved patient self-management and clinical outcomes in conditions such as diabetes [5,6].

However, existing literature often examines internet development and digital health adoption separately. Studies on national internet penetration typically focus on access disparities and device use [7,8], while evaluations of online health services are frequently confined to specific clinical settings, patient groups, or short-term periods following policy changes [5,6,9]. A comprehensive, longitudinal perspective that links the foundational trends of general internet accessibility and use with the emergent trajectory of population-level online health service adoption remains lacking. This disparity restricts the capacity of policymakers and health care providers to formulate strategies according to how the development of digital infrastructure directly facilitates or restricts digital health equity and accessibility.

Therefore, to bridge this gap, this study aimed to conduct an integrated 10-year observational analysis (2014-2025) to describe the parallel trends of internet development—encompassing penetration rates, urban and rural distribution, and access devices—and the adoption of online health services among the general population in China.

## Methods

### Data Source

Data were obtained from the official website of the China Internet Network Information Center (CNNIC) from June 2014 to June 2025. This study was conducted in 31 provinces, autonomous regions, and municipalities, excluding the Hong Kong Special Administrative Region, Macao Special Administrative Region, and the province of Taiwan. This study was a secondary analysis of data from the Statistical Survey Report on Internet Development in China, published semiannually by the CNNIC. The CNNIC survey was designed to measure internet access and use patterns among the Chinese population aged 6 years and above. It used a stratified 2-stage random sampling method via computer-assisted telephone interviewing. The survey measured (1) basic internet access (penetration), (2) devices used for access (eg, smartphones and computers), (3) purposes of internet use (including communication, video, online payment, and since 2020, online health services), and (4) user demographics (urban or rural, age, etc). The data were weighted to represent national and provincial estimates.

### Ethical Considerations

This study used data exclusively from the publicly released Statistical Report on Internet Development in China by CNNIC. The data were aggregated and anonymized without any personally identifiable information. The research did not involve direct interaction with human participants and interventions or the collection of sensitive personal information. In accordance with the policies of the institutional review board of Ningbo Medical Center Li Huili Hospital, secondary analysis of publicly available, fully anonymized data are exempt from ethical review (KY2023ML047).

### Definitions

In this study, an internet user was defined as a resident aged 6 years and older who had used the internet in the past 6 months.

The internet penetration rate is defined as the number of internet users divided by the total resident population of the corresponding region (national, urban, or rural), expressed as a percentage.

Rural and urban users refer to internet users who have mainly lived in rural or urban areas of China in the past 6 months.

Data for specific devices (smartphones, desktops, etc) and services (instant messaging, short video, etc) were extracted directly from CNNIC reports.

Online health users were defined as individuals who prefer to search for health care–related activities via the internet. These activities primarily include online medical and follow-up consultations, medication delivery, health management, and health education.

### Statistical Analysis

We conducted a longitudinal descriptive analysis of national aggregated data. All data on user numbers were extracted directly from CNNIC reports without modification. Calculated indicators, specifically internet penetration rates (derived from user numbers and corresponding population estimates), were presented rounded to 1 decimal place. Core variables included total users, penetration rates, and urban-rural distributions from 2014 to 2025. All data were analyzed using descriptive statistics and visualized through time-series plots.

Annual growth rates were calculated as the percentage change in numbers between consecutive time points, using the following formula:

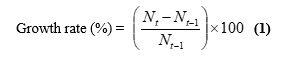



Where *N_t_* represents the number of users at time *t*.

Urban-rural disparity was quantified using both absolute and relative measures:

Absolute gap = urban penetration rate – rural penetration rate **(2)**

Relative gap = urban penetration rate / rural penetration rate **(3)**

The V-shaped trend in online health users (2020-2025) was characterized by comparing user counts at 3 key points—initial (2020), nadir (2023), and final (2025)—and calculating the percentage change between these points. All analyses were performed using R software (version 4.2.1; R Foundation for Statistical Computing) and RStudio (version 2023.06.1; Posit Software, PBC).

## Results

### Trends in Internet Development in China

From 2014 to 2025, an increasing trend was observed in the number of internet users and the internet penetration rate in China. As of June 2025, 1123 million individuals had access to the internet in China, with an internet penetration rate of 79.7% (Table 1).

**Table 1:** Development of the internet in China from 2014 to 2025.

Month and year	Internet users (million), n	Internet penetration rate (%)
June 2014	632	46.9
December 2014	649	47.9
June 2015	668	48.8
December 2015	688	50.3
June 2016	710	51.7
December 2016	731	53.2
June 2017	751	54.3
December 2017	772	55.8
June 2018	802	57.7
December 2018	829	59.6
June 2019	854	61.2
March 2020	904	65.4
June 2020	940	67.0
December 2020	989	70.4
June 2021	1011	71.6
December 2021	1032	73.0
June 2022	1051	74.4
December 2022	1067	75.6
June 2023	1079	76.4
December 2023	1092	77.5
June 2024	1099	78.0
December 2024	1108	78.6
June 2025	1123	79.7

### Internet Users in Urban and Rural Regions

**Figure 1:**
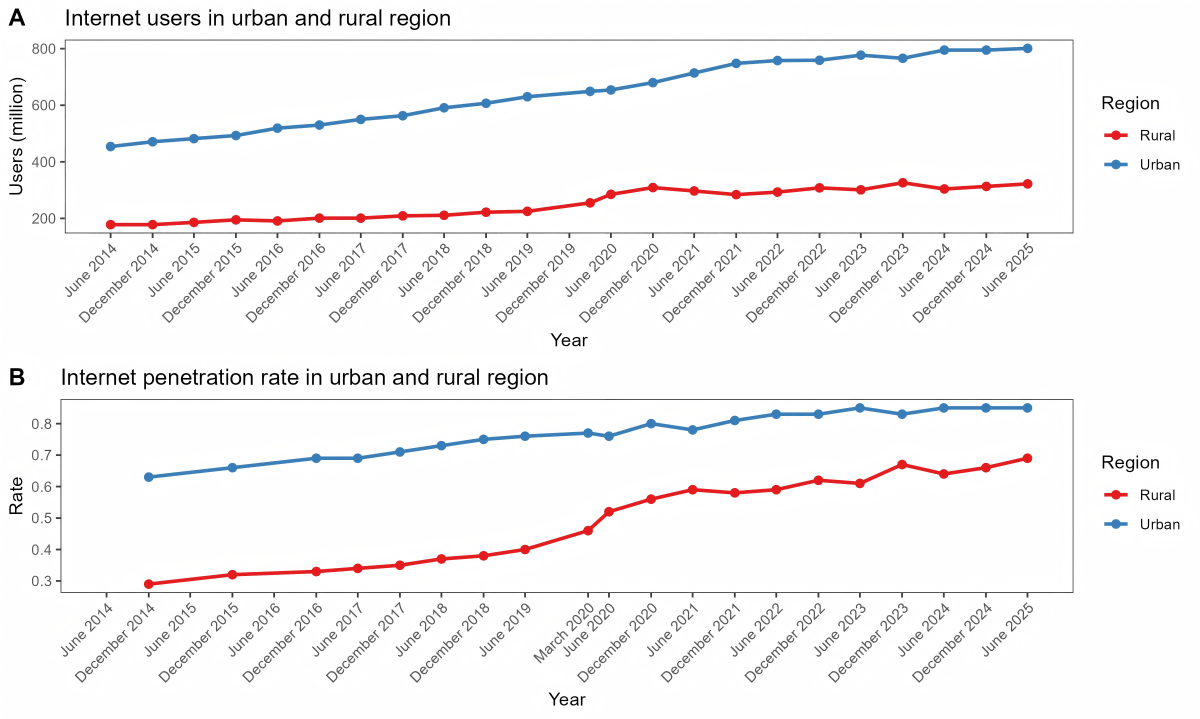
Internet users and the internet penetration rate in urban and rural regions.

In China, the number of internet users has shown an upward trend over the past decade, both in urban and rural areas. By June 2025, there were 801 million internet users in urban areas, corresponding to a penetration rate of 84.9%. The number of internet users in rural areas stood at 322 million, which is less than that in urban areas. Rural areas had a penetration rate of 69.2%. Although there was an obvious disparity in the internet penetration rate between the urban and rural regions, the gap gradually narrowed over time (Figure 1).

### Devices for Internet Access Among Chinese Residents

In China, internet users access the internet via various devices, mainly mobile phones, televisions, desktops, and laptops. From 2014 to 2025, a consistently increasing trend was detected in the number of mobile internet users. In June 2025, smartphones were used by almost all internet users for access. In contrast, desktops and laptops showed an overall declining trend. The use rate of accessing the internet through televisions initially increased and then declined gradually after 2019 (Table 2).

**Table 2:** The penetration rate of different internet access devices in China.

Month and year	Mobile phone (%)	Television (%)	Desktop (%)	Laptop (%)
June 2014	83.4	—^a^	69.6	43.7
December 2014	85.8	15.6	70.8	43.2
June 2015	88.9	16.0	68.4	42.5
December 2015	90.1	17.9	67.6	38.7
June 2016	92.5	21.1	64.6	38.5
December 2016	95.1	25.0	60.1	36.8
June 2017	96.3	26.7	55.0	36.5
December 2017	97.5	28.2	53.0	35.8
June 2018	98.3	29.7	48.9	34.5
December 2018	98.6	31.0	48.0	35.9
June 2019	99.1	33.1	46.2	36.1
March 2020	99.3	32.0	42.7	35.1
June 2020	99.2	28.6	37.3	31.8
December 2020	99.7	24.0	32.8	28.2
June 2021	99.6	25.6	34.6	30.8
December 2021	99.7	28.1	35.0	33.0
June 2022	99.6	26.7	33.3	32.6
December 2022	99.8	25.9	34.2	32.8
June 2023	99.8	26.8	34.4	32.4
December 2023	99.9	22.5	33.9	30.3
June 2024	99.7	25.2	34.2	32.4
December 2024	99.7	25.1	36.2	32.0
June 2025	99.4	24.5	32.3	30.9

^a^Not available.

### Online Services in China

In China, most residents use the internet for communication via instant messaging, browsing videos, short videos, and online payment (Table 3). There has been a substantial rise in user engagement in these activities from 2014 to 2025. Daily communication is the predominant internet service.

**Table 3:** Number of internet users engaging in specific online services in China (2014-2025).

Month and year	Instant message (million), n	Internet video (million), n	Short video (million), n	Online payment (million), n
June 2014	564	439	294	292
December 2014	588	433	313	304
June 2015	606	461	354	359
December 2015	624	504	405	416
June 2016	642	514	440	455
December 2016	666	545	500	475
June 2017	692	565	525	511
December 2017	720	579	549	531
June 2018	756	609	594	569
December 2018	792	612	648	600
June 2019	825	759	648	633
March 2020	896	850	773	768
June 2020	931	888	818	805
December 2020	981	927	873	854
June 2021	983	944	888	872
December 2021	1007	975	934	904
June 2022	1027	995	962	904
December 2022	1038	1031	1012	911
June 2023	1047	1044	1026	943
December 2023	1060	1067	1053	954
June 2024	1078	1068	1050	969
December 2024	1081	1070	1040	1029
June 2025	1093	1085	1068	1022

### Trends in Online Health Use in China

**Figure 2:**
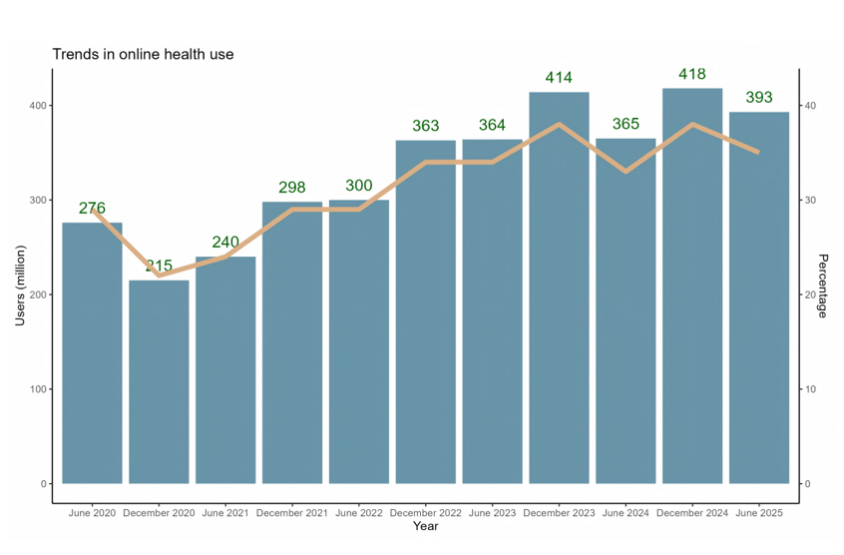
The number of online health-seeking users from 2020 to 2025 in China.

On the basis of data available from 2020 to 2025, online health use in China exhibited a V-shaped pattern during this period. Following a significant increase during the COVID-19 pandemic (2020-2022), user numbers experienced a slight contraction in the first half of 2024 and then rose again toward 2025 (Figure 2).

## Discussion

### Principal Findings

In June 2025, there were 1.123 billion internet users in China. The number of internet users increased dramatically from 2014 to 2025. The number of internet users showed an upward trend in both urban and rural areas in the past decades. Although the gap in internet penetration rate has narrowed between urban and rural areas, it still persists, highlighting the ongoing challenge of the digital divide in China. However, recent research suggests that nationwide investments in information infrastructure, such as the “Broadband China” strategy referenced in our study, can generate a significant “catching-up effect” in rural areas. Specifically, improving information infrastructure has been shown to increase household income more substantially in rural regions, thereby contributing to the reduction of the urban-rural development gap [9]. Empirical studies confirm that improved broadband infrastructure directly stimulates online health service adoption in rural communities, contributing to narrowing the health access gap [10]. This suggests that targeted digital infrastructure policies could be of critical importance for attaining equitable digital inclusion, even when disparities in baseline access continue to exist.

A longitudinal analysis of the penetration rates reveals a trend of convergence. While the absolute gap in internet penetration between urban and rural areas was 40% in 2014, it decreased to 15.7% by June 2025. This narrowing of the penetration rates occurred alongside a period of significant national policy focus on rural digital infrastructure (eg, the “Broadband China” strategy), suggesting that these efforts had a measurable mitigating effect on the digital divide, even as a disparity persists.

After the Chinese socioeconomic reforms, reports focused on the rural-urban disparity in health care [11]. Along with the urban-rural income gap, economic development causes a health care resource gap and health inequality. Ecological and individual-level studies elsewhere have suggested that internet use may be associated with reduced health inequality by promoting health awareness [12]. For instance, evidence suggests that the use of the internet influences mental health inequalities between urban and rural areas [13]. More attention should be paid to older adults. Although their adoption of the internet has witnessed a significant increase, they still constitute a crucial demographic affected by the digital divide. Understanding their use patterns is also crucial for inclusive digital health strategies [14]. Regarding the access devices, smartphones have remained the primary choice, showing a continuous increase in use over the past decade. In contrast to other devices, smartphones have become increasingly popular and affordable. In China, the government launched a series of internet infrastructure projects to lower the threshold for the use of smart terminals [9].

These policy measures have been associated with accelerated internet adoption in rural areas. First, as shown in our results, this contributed to a narrowing of the urban-rural penetration gap over the study period. However, a substantive gap remains (urban: 84.9%; rural: 69.2%) between the regions in June 2025, indicating that continued and targeted efforts are necessary to achieve equitable digital access. Second, smartphones are convenient to carry and provide improved internet capabilities compared with desktop computers and televisions. We will consider “health code” as an example. It was a widely used smartphone-based app to provide precise health protection from 2020 to 2025 nationwide [15]. Third, the government has improved the internet environment by supporting mobile internet access and reducing barriers and costs. The integration of mobile payment platforms (such as Alipay and WeChat Pay) has further eliminated transactional obstacles, rendering online health consumption seamless and more accessible [16]. Finally, the accessibility of diverse mobile internet apps has heightened the inclination to use these services and incorporate them into daily life, appealing to users from conventional industries.

Instant messaging is the predominant form of online digital media use in China. Communication was highly popular among residents across multiple domains, such as the social, occupational, and educational spheres. DingTalk and WeChat are 2 prevalent social media platforms, with WeChat being among the top. DingDing provides official collaboration and organizational management modules. Moreover, large institutions have increased their adoption of enterprise instant messaging, forming partnerships with platforms such as DingDing [17]. QQ can also be used in China, and studies have shown that the use of instant messaging tools, such as QQ, could improve teaching quality in medical imaging technology courses [18]. In addition to instant messaging, internet users of the other 3 types have increased dramatically in the last 10 years, with short videos increasing the most. Several factors could explain these changes. First, there is a strong desire to showcase the achievements of the new era through diverse forms of online audiovisual content, focusing on producing high-quality programs that capture the essence of this era. Additionally, online video platforms are expanding their offerings, including mobile cinema experiences in cars and providing entertainment for people while they travel. For example, Mango TV has partnered with more than 10 car brands to offer video entertainment services on in-vehicle screens, allowing users to enjoy popular programs through the Mango TV client [19]. The dominance of major short video platforms, such as TikTok and Kuaishou, has also led to a differentiated competitive advantage. These platforms have a significantly larger user population than other short video apps, and their respective ecosystems have contributed to increased market concentration. Furthermore, the integration of short video content into e-commerce has improved the e-commerce industry. These platforms have evolved into significant channels for disseminating health information and conducting public health campaigns, particularly influencing health behaviors among younger and middle-aged users [20]. Platforms such as TikTok and Kuaishou actively promote e-commerce marketing and accelerate the integration of online payment services, creating a short video e-commerce ecosystem.

By the end of June 2025, there were 393 million online medical users in China. Among them, most Chinese residents sought medical assistance from an internet hospital. In 2015, the internet hospital emerged in China as a new approach to provide health services and outpatient services, particularly through internet technology [21]. Initially, internet hospitals did not enjoy a high level of public recognition. After the COVID-19 outbreak, the demand for the use of internet medical care reached a peak when offline treatment channels were obstructed. The observed V-shaped trajectory, characterized by a surge during the COVID-19 pandemic peak (2020-2022), a decline in 2024, and a subsequent recovery, reflects the market’s adjustment to a new equilibrium. This pattern aligns with national survey data, which reveals a continuous growth in the use of telemedicine after the COVID-19 pandemic. This indicates a fundamental shift in behavior rather than a temporary surge [22]. The specific and transient decline noted in mid-2024 (June 2024) during this recovery period can potentially be ascribed to multiple converging factors. First, it might indicate a postexpansion consolidation phase. During this phase, the initial high-frequency users from the COVID-19 pandemic period stabilized their use patterns, and the growth resulting from new user acquisition temporarily decelerated. Second, potential alterations in the reimbursement policies for specific types of online consultations by national or regional health care insurers at that time may have temporarily influenced user engagement. The development of national digital health policies has been demonstrated to substantially affect the scale, quality, and user acceptance of internet hospitals over time [23]. Third, by the end of 2023, the COVID-19 pandemic monitoring was not a mandatory requirement; therefore, the use of the internet for scanning codes to monitor SARS-CoV-2 saw a significant decline. Finally, this period coincided with a phase where the market was digesting the initial rapid influx of internet hospitals, and user preferences may have shifted toward more integrated or specialized platforms. This short-term decline underscores that the adoption of digital health services is dynamic and influenced by policy, market maturity, and user behavior, even within a strong positive long-term trend. To further understand the drivers behind these adoption trends, recent syntheses have highlighted a complex interplay of factors influencing digital health uptake in China. Beyond infrastructure access, key determinants include perceived usefulness and ease of use of the platforms, trust in online health information and health care providers, and individuals’ digital literacy and health needs [24]. According to official Chinese data, the number of internet hospitals increased from 1400 in 2018 to 2700 in 2020. Apart from internet hospitals, online medical services and research are still underway. Research has shown that the application of the Internet of Medical Things to patients with interstitial lung disease can enhance individually targeted treatment [25]. Moreover, integrated online-to-offline chronic disease management models, facilitated by these platforms, have demonstrated effectiveness in improving clinical outcomes in primary care settings [26]. Yeh et al [25] suggested that web-based diabetes prevention programs can delay the onset of type 2 diabetes, providing culturally and linguistically appropriate interventions for Chinese Americans. It should also be noted that although access is on the rise, inequalities in the capacity to proficiently use digital tools for health purposes (the “second-level digital divide”) persist. These inequalities are manifested in and may intensify the preexisting socioeconomic and spatial disparities in the actual use and perceived quality of online health services, as demonstrated by studies on internet hospitals [27].

It is important to acknowledge a key limitation inherent in the aggregate CNNIC data used in this analysis—while they capture whether individuals used online health services within the past 6 months, they cannot distinguish between frequent, infrequent, or 1-time users. This lack of granularity regarding use intensity and user retention influences the interpretation of the observed V-shaped trend. For instance, the post-2023 rebound might reflect a consolidation toward a core group of repeat users who have integrated online health services into their regular care routines rather than a broad re-engagement of the general population. Conversely, the 2023 dip could represent a shedding of less-engaged or trial users while a stable base of dedicated users persisted. Therefore, while the trend indicates overall adoption dynamics, it does not elucidate whether growth is driven by deepening engagement among existing users or expansion to new ones. Future studies incorporating individual-level longitudinal data on use frequency and continuity are needed to disentangle these patterns and better understand the maturation of the digital health market in China.

This study still has other limitations. First, while aggregate internet use trends are based on complete data (2014-2025), detailed metrics on online health users are only available from 2020 onward, which restricts our long-term trend analysis for this specific sector. We have addressed this by clearly framing the online health analysis within the 2020 to 2025 period and by performing sensitivity analyses on the core internet adoption trends to confirm their robustness. Second, as an ecological study using aggregate administrative data, it cannot account for individual-level confounding factors or establish causality between internet development and health service adoption. The observed trends, while nationally representative, should be interpreted as descriptive associations that generate hypotheses for future causal investigation. Despite these limitations, this analysis provides a comprehensive and robust overview of the 10-year evolution of internet infrastructure and adoption patterns in China, establishing a reliable baseline for future investigations.

### Conclusions

This study demonstrates that the internet in China continued to develop significantly and steadily from 2014 to 2025, with an increasing number of internet and online medical users in both urban and rural areas. Smartphones remained the primary device for accessing the internet. The internet and short videos increased significantly, nearly surpassing instant messaging in user numbers. Notably, the observed V-shaped trajectory of online health adoption—marked by a COVID-19 pandemic–driven surge followed by consolidation and recovery—suggests that the digital health market is maturing through a phase of user-base stabilization. In light of this finding, we recommend that policymakers and health care providers focus not only on promoting initial adoption but also on developing mechanisms to retain users and ensure consistent service quality. Strategies should be made, including integrating health services into highly engaging platforms (such as short-video apps), improving user experience, and cultivating trust in online care, which may contribute to consolidating the recovery phase of the V-shaped trajectory and averting future downturns. To maximize the impact, such strategies should be based on a comprehensive understanding of the evolving digital health ecosystem, which includes infrastructure, user behavior, and policy enablers, as synthesized in recent national-level analyses [28].

## Data Availability

The datasets generated or analyzed during this study are available from the corresponding author on reasonable request.

## Abbreviations

CNNIC: China Internet Network Information Center
